# *Zingiber zerumbet* L. (Smith) extract alleviates the ethanol-induced brain damage via its antioxidant activity

**DOI:** 10.1186/s12906-018-2161-5

**Published:** 2018-03-20

**Authors:** Asmah Hamid, Farah Wahida Ibrahim, Teoh Hooi Ming, Mohd Nazir Nasrom, Norelina Eusoff, Khairana Husain, Mazlyzam Abdul Latif

**Affiliations:** 1 Biomedical Science Programme, Centre of Health & Applied Sciences, Faculty of Health Sciences, Universiti Kebangsaan Malaysia, Jalan Raja Muda Abdul Aziz, 50300 Kuala Lumpur, Malaysia; 2 Faculty of Pharmacy, Universiti Kebangsaan Malaysia, Jalan Raja Muda Abdul Aziz, 50300 Kuala Lumpur, Malaysia

**Keywords:** *Zingiber zerumbet*, Ethanol, Brain damage, Antioxidant, Oxidative stress

## Abstract

**Background:**

*Zingiber zerumbet* (L.) Smith belongs to the Zingiberaceae family that is widely distributed throughout the tropics, particularly in Southeast Asia. It is locally known as ‘*Lempoyang*’ and traditionally used to treat fever, constipation and to relieve pain. It is also known to possess antioxidant and anti-inflammatory activities. Based on these antioxidant and anti-inflammatory activities, this study was conducted to investigate the effects of ethyl-acetate extract of *Z. zerumbet* rhizomes against ethanol-induced brain damage in male Wistar rats.

**Method:**

Twenty-four male Wistar rats were divided into four groups which consist of normal, 1.8 g/kg ethanol (40% *v*/v), 200 mg/kg *Z. zerumbet* extract plus ethanol and 400 mg/kg *Z. zerumbet* plus ethanol. The extract of *Z. zerumbet* was given once daily by oral gavage, 30 min prior to ethanol exposure via intraperitoneal route for 14 consecutive days. The rats were then sacrificed. Blood and brain homogenate were subjected to biochemical tests and part of the brain tissue was sectioned for histological analysis.

**Result:**

Treatment with ethyl-acetate *Z. zerumbet* extract at 200 mg/kg and 400 mg/kg significantly reduced the level of malondialdehyde (MDA) and protein carbonyl (*p* < 0.05) in the brain homogenate. Both doses of extracts also significantly increased the level of serum superoxide dismutase (SOD), catalase (CAT) and glutathione peroxidase (GPx) activities as well as glutathione (GSH) level (*p* < 0.05). However, administration of ethyl-acetate *Z. zerumbet* extract at 400 mg/kg showed better protective effects on the ethanol-induced brain damage as shown with higher levels of SOD, CAT, GPx and GSH in the brain homogenate as compared to 200 mg/kg dose. Histological observation of the cerebellum and cerebral cortex showed that the extract prevented the loss of Purkinje cells and retained the number and the shape of the cells.

**Conclusion:**

Ethyl-acetate extract of *Z. zerumbet* has protective effects against ethanol-induced brain damage and this is mediated through its antioxidant properties.

**Graphical abstract:**

Z. zerumbet extract protects against ethanol-induced brain damage via its antioxidant properties
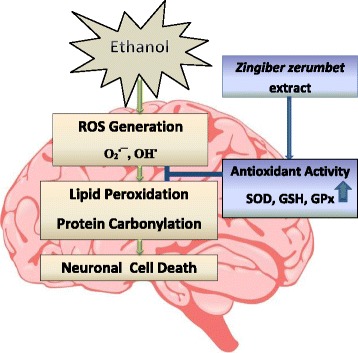

## Background

Alcohol is a commonly abused substance worldwide that generates reactive oxygen species (ROS) whether it is consumed acutely or chronically [1]. Alcohol can easily cross the cell membranes, including the blood-brain barrier. Although it is metabolized mainly in the liver, it also causes toxic effects to the brain [2]. As the brain is rich in lipid content, it has high oxygen demand and lacks antioxidant activities thus very susceptible to oxidative stress as the brain easily undergo redox-reaction leading to radicals formation [3].

Alcohol causes oxidative damage by producing superoxide, hydroxyl and hydroethyl radicals which may contribute to lipid peroxidation, protein carbonyl formation and DNA damage [4]. Furthermore, it can disrupt the antioxidant system to cause oxidative stress [5] which eventually leads to neuronal cell death [6]. In normal condition, ROS and antioxidants are in a balanced state. Oxidative stress occurs when oxidants and antioxidants are in imbalance [7] either caused by excessive ROS or antioxidant system dysfunction [8]. Antioxidants are used to scavenge ROS [9].

Ethanol consumption has been associated with brain damage. Necropsy examinations of chronic alcoholics have shown a variety of structural and functional alterations in the neurons [10]. One brain region that is particularly sensitive to early ethanol exposure is the cerebellum. Previous study was found that 2 days of highly condensed alcohol exposure during neonatal brain growth resulted in Purkinje cell loss [11].

Zingiberaceae family is distributed widely in Asia where 50 genera and more than 1000 species can be found in this family including *Curcuma longa* which produces curcumin. Curcumin has been reported to have high antioxidant activities that protect the brain against ethanol-induced brain damage [1, 12]. *Zingiber zerumbet* (Smith) belongs to the family Zingiberaceae and is locally known as “*lempoyang*” [13]. *Z. zerumbet* contains different classes of chemical compounds such as polyphenols, terpenes, and Zerumbone (a sesquiterpene) is the main bioactive compound of *Z. zerumbet* [14].

Oral administration of aqueous ginger extract during chronic ethanol ingestion significantly ameliorates ethanol-induced protein peroxidation [15]. The antioxidant protective properties of polyphenol and flavonoid components of the extract possibly increase the antioxidative defence mechanism of the cells. *Zingiber officinale* extract was a potential brain tonic to enhance cognitive function for ethanol-related cognitive deficits [16]. The plant extract was found to exhibit protective capability and cognitive enhancing properties in ethanol-induced rats. It was also shown that *Z. officinale* at the dose of 200 mg/kg body weight could protect the brain against ischemic brain damage and reduced cognitive deficits in a rat model of focal cerebral ischemia induced by the occlusion of the right middle cerebral artery. This family of ginger was found to reduce the brain infarct volume and decrease oxidative stress in the cerebral cortex and hippocampus. Neuroprotective effect of *Z. officinale* extract might be related to its antioxidant effect.

Our previous study demonstrated that administration of *Z. zerumbet* ethyl acetate extract at 200 or 400 mg/kg displayed protective effects and antioxidant activities against paracetamol-induced nephrotoxicity. This was shown by increased in both antioxidant SOD and GSH level and reduction of both homogenate and plasma MDA, plasma protein carbonyl, and renal advanced oxidation protein product (AOPP) than those in the paracetamol induced group [17].

Another study on oral administration of ethyl acetate extract of *Z. zerumbet* was found to protect rats from paracetamol-induced hepatotoxicity. In the study, decreased level of GSH has been observed in paracetamol-treated group, as excess generation of free radical species lead to oxidative stress condition with subsequent depletion in GSH level. However, *Z. zerumbet* administration appeared to cause less oxidative stress condition as its ability to reduce protein and lipid oxidation and increase the antioxidant defense mechanism. Remarkable protective effects were observed at 400 mg/kg as compared to a lower dose of 200 mg/kg of the extract. This indicates that the amount of antioxidant compounds present in *Z. zerumbet* contributes significantly to its antioxidant property [18].

The rhizomes of *Z. zerumbet* are used to treat ulcerative colitis [19] to cure dyspepsia wound, hemorrhoids, and stomach discomfort [20]. *Z. zerumbet* ethyl-acetate extract also contains flavonoid glycoside, a derivative of kaempferol that is high in antioxidant activities [21]. However, to date, evidence on the neuroprotective effects of *Z. zerumbet* rhizomes is still lacking. Thus, the aim of this study is to investigate the protective effects of the ethyl-acetate extract of *Z. zerumbet* rhizomes against ethanol-induced brain damage in male Wistar rats.

## Methods

### Plant extraction

*Zingiber zerumbet* rhizomes were supplied from herbs farm (Kizaherbs Sdn Bhd) in Temerloh, Pahang, Malaysia. The plant was identified by a botanist and herbarium curator, Mr. Ahmad Damanhuri Mohamad from School of Environmental and Natural Resource Sciences and deposited at the Faculty of Science and Technology Herbarium, Universiti Kebangsaan Malaysia. It was given a voucher specimen number UKMB-29952. The air dried rhizomes of *Z. zerumbet* were soaked in *n*-hexane for 72 h at room temperature. This process was repeated three times. The rhizomes then underwent similar process with ethyl acetate followed by methanol. Each crude extract of hexane, ethyl-acetate and methanol was filtered and evaporated using rotary evaporator. The yielded extracts were subjected to dryness in a fume hood. The crude extracts were then stored at 4 °C until tested for bioassay. Prior to use, ethyl-acetate extract of *Z. zerumbet* was dissolved in di-methyl sulfoxide (DMSO) and diluted in phosphate buffered saline (PBS) pH 7.4.

### Animals and treatment

A total of 24 male albino Wistar rats were obtained from Laboratory Animal Resources Unit, Universiti Kebangsaan Malaysia (UKM). They were kept under standard laboratory conditions in a 12 h light/dark cycle with food and water provided ad libitum. The experiment involving the animals was approved according to animal handling guidelines provided by the UKM Animal Ethics Committee (FSK/BIOMED/2012/ASMAH/21-NOV/475-NOV-2012-JUNE-2013).

The animals were randomly divided into four groups with six rats in each group. Rats in Group 1 received normal saline (normal), Group II were ethanol-induced group where ethanol was administrated intraperitoneally at the dose of 1.8 g/kg^− 1^(40% *v*/v). Group III and IV were given the extract of *Z. zerumbet* via oral gavage at the doses of 200 mg/kg and 400 mg/kg respectively, 30 min prior to ethanol exposure, for 14 consecutive days. After 14 days, the rats were anaesthetized with a cocktail of ketamine, xylazil and zoletil (0.5 ml/kg body weight) given intramuscular [22] and sacrificed through decapitation according to approved guidelines from UKM Animal Ethics Committee.

### Sample preparation

Blood was collected by cardiac puncture in plain tubes and centrifuged at 3000 rpm for 10 min to obtain serum. Serum samples were stored at − 20 °C for subsequent biochemical analysis. Brain samples were washed with normal saline and homogenized in cold 1.15% potassium chloride (KCI) (3 ml/g) using Ultra Turrax T25 homogenizer (IKA, Germany).

### Biochemical analysis

Malondialdehyde (MDA) level is an index to determine lipid peroxidation process (Ledwozyw et al. 1986) [23]. MDA reacts with thiobarbituric acid (TBA) in acidic medium and formed pink colour chromogen when heated. Butanol was used to extract this chromogen and its absorbance was measured spectrophotometrically at the wavelength of 532 nm.

Protein carbonyl level was determined according to a method as described by Levine [24] based on the reaction of carbonyl compounds with 2, 4-dinitrophenyl hydrazine (DNPH). Trichloroacetic acid (TCA) was used to precipitate the protein. The precipitated protein was centrifuged to form pellet. The procedure was followed by two steps of washing using ethanol/ethyl-acetate that would remove lipids from the protein pellet. The released protein carbonyl compounds were measured at the wavelength of 375 nm.

SOD activity was assayed based on the procedure described by Beyer & Fridovich [25]. This assay was based on the reduction of nitro blue tetrazoleum (NBT) by superoxide anion to form diformazan and the absorbance was measured with a spectrophotometer at 560 nm wavelength. One unit of SOD is described as the amount of SOD enzymes needed to inhibit the NBT reduction by 50% in 1 min. CAT activity was measured by Aebi method [26] and its principle was based on the ability of CAT enzymes to catalyze the reduction of H_2_O_2_ to O_2_. Decomposition of H_2_O_2_ by CAT enzymes was measured at 240 nm wavelength.

GPx enzyme activity was assayed according to the procedure described by Paglia & Valentine [27] based on the oxidation rate of glutathione (GSH) by H_2_O_2_ in the presence of GPx. Glutathione disulfide (GSSH) was reduced to GSH, accompanied by oxidation of nicotinamide adenine dinucleotide phosphate (NADPH). NADPH oxidation was measured at 340 nm wavelength and one unit of GPx is interpreted as one mole of NADPH required to be oxidized to NADP. GSH was quantified according to the protocols as described by Ellman [28]. GSH oxidation by 5, 5-dithiobis-2-nitrobenzoic (DTNB) reagent to form yellow-coloured complexes (TNB) was measured at the wavelength of 420 nm.

### Histological analysis

Brain tissues samples were fixed in 10% formalin and dehydrated in an ascending series of alcohol concentration (50% to 100%) and then embedded in paraffin blocks. The blocks were cut into 5 μm sections using a microtome, fixed on slides followed with hematoxylin and eosin (H&E) staining. The prepared slides were observed under light microscope (Olympus BX41 Japan).

### Statistical analysis

Statistical analysis was performed by using Statistical Package for the Social Sciences (SPSS) version 19. Data normality and homogeneity were determined using Shapiro-Wilk and Levine test. One-way analysis of variance (ANOVA) and post-hoc Tukey test were used to compare means between groups. Data were expressed as mean ± standard error of mean (SEM).

## Results

### Effects of *Z. zerumbet* extract on oxidative stress markers

#### MDA level

**Fig. 1:**
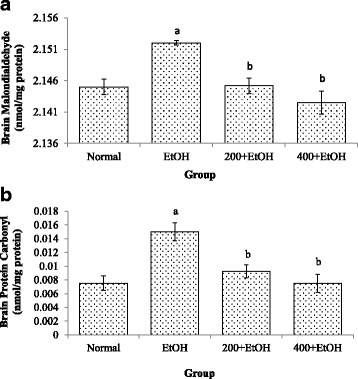
**a** Effects of *Z. zerumbet* extract on malondialdehyde level. Values are expressed as mean ± SEM. “a” significant at F(5,18) = 16.978 *p* < 0.05 compared to normal group; “b” significant at *p* < 0.05 compared to ethanol group (*N* = 6). (EtOH: ethanol; 200 + EtOH: 200 mg/kg extract + ethanol; 400 + EtOH: 400 mg/kg extract + ethanol). **b** Effects of *Z. zerumbet* extract on protein carbonyl level. Values are expressed as mean ± SEM. “a” significant at F(5,18) = 16.978 *p* < 0.05 compared to normal group; “b” significant at *p* < 0.05 compared to ethanol group (*N* = 6). (EtOH: ethanol; 200 + EtOH: 200 mg/kg extract + ethanol; 400 + EtOH: 400 mg/kg extract + ethanol)

A significant increased (*p* < 0.05) level of MDA was observed in brain samples of ethanol-induced group as compared to the normal group. Supplementation with *Z. zerumbet* extracts at 200 mg/kg and 400 mg/kg significantly decreased (*p* < 0.05) MDA levels compared to the ethanol-induced group (Fig. 1a).

#### Protein carbonyl level

Administration of ethanol alone caused a significant increase (*p* < 0.05) in protein carbonyl level in the brain samples compared to the normal group. However, protein carbonyl levels were found to be significantly lower (*p* < 0.05) in both *Z. zerumbet* treated group (200 mg/kg and 400 mg/kg) as compared to the ethanol-induced group (Fig. 1b).

### Effects of *Z. zerumbet* extract on antioxidant status (SOD, CAT, GPx & GSH)

**Fig. 2:**
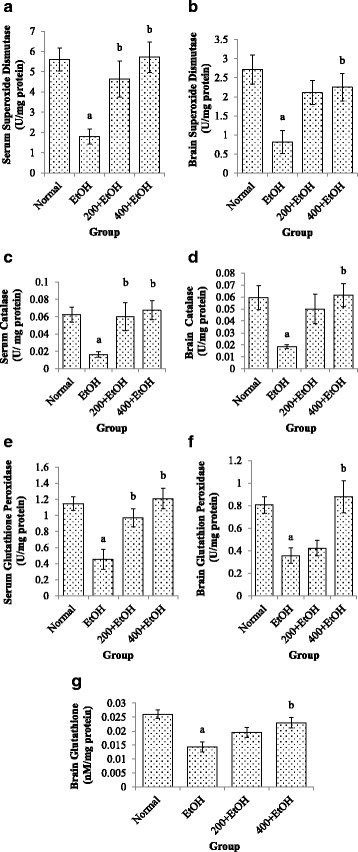
Effects of *Z. zerumbet* extract on serum superoxide dismutase (**a**), brain superoxide dismutase (**b**), serum catalase (**c**), brain catalase (**d**), serum glutathione peroxidase (**e**), brain glutathione peroxidase (**f**) activities and brain glutathione level (**g**). Values are expressed as mean ± SEM. “a” significant at F(5,18) = 16.978, *p* < 0.5 compared to normal group; “b” significant at *p* < 0.05 compared to ethanol group. (*N* = 6). (EtOH: ethanol; 200 + EtOH: 200 mg/kg extract + ethanol; 400 + EtOH: 400 mg/kg extract + ethanol)

Administration of ethanol for 14 days induced oxidative stress as evidenced by a significant reduction (*p* < 0.05) of SOD, CAT and GPx enzyme activities (Fig. 2a-f) in both serum and brain samples. Treatment with *Z. zerumbet* extract at 200 mg/kg and 400 mg/kg significantly increased (*p* < 0.05) the SOD, CAT and GPx enzyme activities in serum samples. For the brain samples, only 400 mg/kg *Z. zerumbet* extract showed significant increased (*p* < 0.05) in the activities of these three enzymes as compared to the ethanol-induced group. Besides that, GSH level was significantly decreased (*p* < 0.05) in ethanol-induced group as compared to the normal group (Fig. 2g). For 400 mg/kg *Z. zerumbet* treated groups, there was a significant increment (*p* < 0.05) in the GSH level but 200 mg/kg *Z. zerumbet* treated group showed no significant difference (*p* > 0.05) compared to the ethanol group.

### Effects of *Z. zerumbet* extract on histological features of the brain

**Fig. 3:**
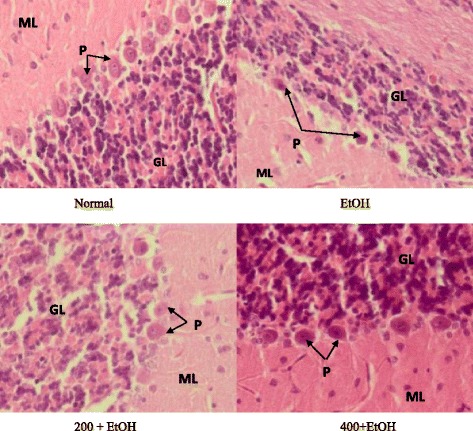
Brain sections (cerebellum) from each group (magnification × 40). Note the number of Purkinje cells. (P- Purkinje cell; ML-Molecular layer; GL-Granular layer). (*N* = 6). (EtOH: ethanol; 200 + EtOH: 200 mg/kg extract + ethanol; 400 + EtOH: 400 mg/kg extract + ethanol)

**Fig. 4:**
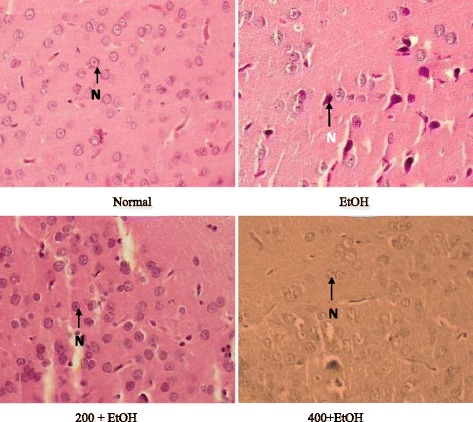
Brain sections (cerebral cortex) from each group (magnification X40). Note the number and shape of neuron cells. (N-Neuron cell). (*N* = 6). (EtOH: ethanol; 200 + EtOH: 200 mg/kg extract + ethanol; 400 + EtOH: 400 mg/kg extract + ethanol)

Microscopic structures of the brain (cerebellum and cortex cerebrum) from normal, ethanol, 200 mg/kg + ethanol, and 400 mg/kg + ethanol groups were observed as presented in Figs. 3 and 4. Figure 3a showed that no histological changes were observed in the normal, 200 mg/kg + ethanol, and 400 mg/kg + ethanol groups as evidenced by normal Purkinje cells in the cerebellum (Fig. 3) and normal neuron cells in the cerebral cortex (Fig. 4). On the other hands, brain sections from ethanol-induced group showed that the distance between 2 Purkinje cells was large as compared to normal group. Neuron cells in in the cerebral cortex undergo necrosis with pyknotic characteristic such as darken nucleus and lost its morphology.

## Discussion

Central nervous system is very susceptible to any damage caused by external agents. Brain is rich in polyunsaturated fatty acids and iron. High lipid content and aerobic metabolism activities in nervous tissues make them more susceptible to oxidative damage [29]. Furthermore, lack of antioxidant activities (CAT, SOD and GPx) in the brain also make it more susceptible to damage caused by ROS. Oxidative stress is caused by an imbalance of redox state in the cell, either excess ROS or defect in the antioxidant system [8]. Oxidative stress induced by short-term alcohol-intake [30] ultimately lead to neuronal cell death [6]. Ethanol dosage used in this study was selected based on the previous study [12] that showed protective effects of *Coscinium fenestratum* against ethanol-induced neurodegeneration in adult rat brain.

Cerebellum is responsible for coordinating muscle activities and maintaining body posture and balance. Cerebral cortex consists of three levels; molecular layer, granular layer and a layer of Purkinje. In addition, cerebral cortex is the outermost layer of the cerebrum and contains billion of neurons that play a role in memory, intelligence and muscle movement [31]. Neuronal cells are highly dependent on glucose for ATP generation and the production of ROS as a product of oxidative phosphorylation in the mitochondria. The resulting ROS production causes the imbalance between antioxidant and oxidative stress, that eventually will lead to neuronal cell damage [32]. That is why both cerebral cortex and cerebellum are the main target in this study.

Elevation of MDA level showed that alcohol produced ROS and caused oxidative stress in the brain. The ability of alcohol to induce lipid peroxidation is associated with direct or indirect damage caused by ethanol or acetaldehyde. Lipids are biomolecules that often become the target of oxidative stress and its oxidation will produce many secondary products such as MDA. Increased in the lipid peroxidation is represented by an increase in the MDA concentration, which is a good biomarker for oxidative stress in the brain [3]. Besides that, protein is also one of the targets of ROS and its oxidation will cause protein to lose its functions. Protein carbonyl level is an indicator of protein damage that can be measured after alcohol consumption.

In contrast, treatment with *Z. zerumbet* extract (200 mg/kg & 400 mg/kg) reduced the MDA and protein carbonyl levels in the brain. The ability of *Z. zerumbet* to protect brain tissues from lipid peroxidation and protein oxidation is in agreement with Hamid et al. [18] and Abdul Hamid et al. [17] studies, where supplementation of *Z. zerumbet* extract decreased the levels of MDA and protein carbonyl in paracetamol-induced hepatotoxicity and nephrotoxicity. These studies also supported that *Z. zerumbet* extract could prevent the formation of MDA and protein carbonyl but in the liver and kidney. *Z. zerumbet* ethyl-acetate extract contained flavonoid glycoside, one of kaempferol derivative that was characterized by radical scavenging activity and lipid peroxidation inhibition [21]. Zerumbone, the active compound of *Z. zerumbet* rhizomes protected the kidney against cisplastin-induced nephrotoxicity by blocking the MDA formation [33].

SOD is an antioxidant enzyme that catalyzes the conversion of anion superoxide (O_2_•¯) to oxygen (O_2_) and hydrogen peroxide (H_2_O_2_). Reduction of SOD activity in the brain induced by ethanol in this study may be due to the generation of excessive anion, (O_2_^•^¯) that causes this enzyme to become inactivated. CAT enzyme involves in the conversion of H_2_O_2_ to H_2_O and O_2._ The current study showed that ethanol decreased CAT activity in the brain after 14 days of alcohol exposure. This suggest that the decreased in the CAT activity may be due to the loss of nicotinamide adenine dinucleotide phosphate (NADPH), anion (O_2_^•^¯) production and lipid peroxidation [34].

This study showed that *Z. zerumbet* extract administration increased the activities of SOD and CAT in the serum and brain homogenate. Zerumbone, an active compound isolated from rhizomes of *Z. zerumbet* could inhibit the production of anion O_2_^•^¯ in both NADPH oxidase and xanthine oxidase in acute promyelocytic leukemia cells and AS52 Chinese hamster ovary cells, respectively [35]. Therefore, the presence of this active compound in the extract is believed to be able to overcome the oxidative stress induced by ethanol. Furthermore, the antioxidant activity of *Z. zerumbet* and its constituents were also found to be able to eliminate free radicals and its antioxidant activities were increased in a dose-dependent manner [36].

GPx enzyme is responsible for detoxifying H_2_O_2_ by converting it to H_2_O in which GSH acts as an electron donor in this reaction [37]. On the other hand, GSH is a non-enzymatic antioxidant that serves to prevent oxidative damage caused by ROS. Impairment in GSH cellular defence mechanisms leads to the development of oxidative stress [38]. It also acts as a nucleophilic scavenger of toxic compounds and as a substrate in the reaction of other antioxidants (GPx uses GSH as a cofactor) [39]. In the current study, ethanol administration decreased GPx activity and GSH level both in serum and brain homogenate. ROS produced by ethanol exposure for 14 days probably deactivated both GPx and GSH enzyme activities [34].

However, administration of *Z. zerumbet* extract appeared to reduce oxidative stress induced by ethanol as evidenced by increased level of GSH in the brain. Zerumbone indirectly induced the GSH synthesis and provided intracellular protection mechanism to remove free radicals from toxic agents [33]. Thus, it could be speculated that *Z. zerumbet* extract may induce GSH synthesis by protecting the brain against ethanol-induced damage through similar mechanism. Zerumbone isolated from the rhizomes of *Z. zerumbet* could reduce the accumulation of MDA and increased GSH and GSH reductase (GR) activities against UVB-induced photokeratitis in mice [40] . In addition, Hamid et al. [18] and Abdul Hamid et al. [17] also reported that ethyl-acetate extract of *Z. zerumbet* could increase SOD and GSH activities in paracetamol-induced hepatotoxicity and nephrotoxicity in rats. Thus, *Z. zerumbet* extract is proven not only to increase the antioxidant enzyme activities in the liver and kidney but also in the brain.

*Z. zerumbet* extract at 400 mg/kg dose gave a better protective effect against ethanol-induced brain damage and this may be due to the high amount of zerumbone present in the extract. Increased SOD, CAT and GPx activities in this study may relate to the fact that the high antioxidant activities in *Z.zerumbet* extract induced endogenous antioxidant thus reduced the free radical activity [41]. In addition, an increase in the antioxidant activity can be described as an adaptive response to excessive ROS [42]. Although enzymatic activities of SOD, CAT, GPx and GSH in the *Z. zerumbet* treated group (200 mg/kg) were increased, but the increment were non-significant. Most probably, the 200 mg/kg of *Z. zerumbet* extract was insufficient to overcome the ROS generated by the ethanol induction. For the histological observation of the cerebellum, this study showed that the distance between any two Purkinje cells was larger in ethanol-induced rats, but a detailed and systematic calculation of the distance is required to be carried out to elucidate this issue.

Necrosis was observed in neuron cells (cerebral cortex) of ethanol-induced group with pyknotic characteristic such as darken nucleus and loss of its normal shape under light microscope. This characteristic is in agreement with Phachonpai et al. [12] that demonstrated ethanol administration at the dose of 1.8 g/kg via intraperitoneal for 14 days caused neurodegeneration in the cerebral cortex. Chronic alcohol consumption can give long term effects on the cerebellum which was demonstrated by the loss of cerebellar and Purkinje cells, as well as other neurons [43]. A qualitative study found that there was a loss of Purkinje cells in the vermis with a reduction by 43% on average [44]. One study also reported ethanol administration did induced brain damage [45]. Significant brain damage in adults is found in limbic association regions and in these regions, dark cell degeneration, a necrotic cell death is the predominant form of neuronal death [46]. In contrast, concurrent treatment with *Z. zerumbet* extract, protected cerebellum and cerebral cortex against ethanol-induced brain damage as evidenced by no differences in the morphology of Purkinje cells and neuron cells in both *Z. zerumbet* extract treated rats (200 mg/kg and 400 mg/kg) compared with the normal rats.

## Conclusion

Based on this finding, *Z. zerumbet* ethyl-acetate extract is proven to possess protective effects against ethanol-induced brain damage in Wistar rats by decreasing lipid and protein oxidation as well as increasing antioxidant activities. This suggest that *Z. zerumbet* has the potential as a neuroprotective agent. Further studies are needed to explore its neuroprotective underlying mechanism.

## Abbreviations

CAT: Catalase
DMSO: Di-methyl sulfoxide
DNPH: Dinitrophenyl hydrazine
GPx: Glutathione peroxidase
GSH: Glutathione
GSSH: Glutathione disulfide
KCI: Potassium chloride
MDA: Malondialdehyde
NADPH: Nicotinamide adenine dinucleotide phosphate
NBT: Nitro blue tetrazoleum
PBS: Phosphate buffered saline
ROS: Reactive oxygen species
SOD: Superoxide dismutase
TBA: Thiobarbituric acid
TCA: Trichloroacetic acid
